# 1573. Impact of the COVID-19 Pandemic on Antibiotic Use among Hospitalized Adults in Argentina, Brazil, and Chile

**DOI:** 10.1093/ofid/ofac492.102

**Published:** 2022-12-15

**Authors:** Twisha S Patel, Olivia L McGovern, Garrett W Mahon, Icaro Boszczowski, Jose M Munita, Maria I Garzon, Flavio Lipari, Matias Salomao, Giovanna Marssola, Bruno Tavares, Debora Francisco, Alessandra P A Gurgel, Tiago Arantes, Andrea Bori, Cassimiro Nogueira, Anne Peters, Maria Spencer, Cristian Orellana, Mario Barbe, Analía Roldán, Constanza Lopez, Josefina Bortoletto, Stacie C Stender, Fernanda C Lessa

**Affiliations:** Chenega Enterprise Systems and Solutions (CHESS) / Centers for Disease Control and Prevention, Ann Arbor, MI; U.S. CDC, Atlanta, Georgia; Centers for Disease Control and Prevention, Atlanta, Georgia; Hospital Alemão Oswaldo Cruz, São Paulo, Sao Paulo, Brazil; Facultad de Medicina Clinica Alemana Universidad del Desarrollo, Santiago, Region Metropolitana, Chile; Hospital Privado Universitario de Córdoba, Cordoba, Cordoba, Argentina; Hospital Dr. Angel Ferreyra, Cordoba, Cordoba, Argentina; Hospital das Clinicas da Universidade de São Paulo, Sao Paulo, Sao Paulo, Brazil; Hospital Alemão Oswaldo Cruz, São Paulo, Sao Paulo, Brazil; Hospital das Clínicas Faculdade de Medicina da USP and Hospital Alemão Oswaldo Cruz, São Paulo, Sao Paulo, Brazil; Hospital Alemão Oswaldo Cruz, São Paulo, Brazil, São Paulo, Sao Paulo, Brazil; Hospital Alemão Oswaldo Cruz, São Paulo, Brazil, São Paulo, Sao Paulo, Brazil; Hospital das Clínicas da Faculdade de Medicina da Universidade de São Paulo, São Paulo, Sao Paulo, Brazil; Instituto do Coração HCFMUSP, São Paulo, Sao Paulo, Brazil; Hospital Alemão Oswaldo Cruz, São Paulo, Sao Paulo, Brazil; Instituto de Ciencias e Innovación en Medicina, Santiago, Region Metropolitana, Chile; Instituto de Ciencias e Innovación en Medicina, Santiago, Region Metropolitana, Chile; Hospital Padre Hurtado, Santiago, Chile, Santiago, Region Metropolitana, Chile; Clínica Alemana, Universidad del Desarrollo, Santiago, Region Metropolitana, Chile; Hospital Privado Universitario de Córdoba, Córdoba, Argentina, Cordoba, Cordoba, Argentina; Hospital Privado Universitario de Córdoba, Córdoba, Argentina, Cordoba, Cordoba, Argentina; Hospital Privado Universitario de Córdoba, Córdoba, Argentina, Cordoba, Cordoba, Argentina; Jhpiego & Johns Hopkins University, Baltimore, Maryland; CDC, Atlanta, Georgia

## Abstract

**Background:**

Reports showing high rates of antibiotic use (AU) in patients with coronavirus disease 2019 (COVID-19) despite low rates of secondary bacterial infection have emerged from various countries across the globe. We evaluated the impact of the COVID-19 pandemic on AU in healthcare facilities (HCFs) in Argentina, Brazil, and Chile.

**Methods:**

We conducted an ecologic evaluation of AU in inpatient adult acute care wards (excluding maternity wards) in 6 HCFs in Argentina, Brazil, and Chile; 2 HCFs per country. AU data for intravenously administered antibiotics commonly used to treat respiratory infections were collected from pharmacy dispensing records and aggregated to monthly defined daily dose (DDD)/1000 patient days. Graphs were created to depict AU and COVID-19 discharges over time throughout the 36-month study period (03/2018–02/2021). Relative changes in AU for all antibiotics combined and specific classes were calculated by comparing median AU for the 24-month pre-pandemic period (3/2018–2/2020) with the 12-month pandemic period (3/2020–2/2021). Only statistically significant differences (P< 0.05) determined by the Wilcoxon signed-rank test are reported.

**Results:**

Compared to the pre-pandemic period, the use of all included antibiotics combined increased in 4/6 HCFs (6.7–35.1%). In the 4 HCFs that experienced increases in AU, Figure 1 shows that use was high during months when COVID-19 patient surges occurred. In 3/4 of these HCFs, AU remained high despite significant decreases in COVID-19 discharges. Ceftriaxone use increased in 2/6 HCFs (27.1–51.6%). Use of β-lactam antibiotics with activity against *Pseudomonas aeruginosa* increased in 3/6 HCFs (31.3–82.5%) and decreased in 1/6 HCFs (-18.9%). Vancomycin and linezolid use increased in 3/6 HCFs (36.9–77.1%).

**Conclusion:**

Increases in AU among hospitalized adults were observed in 4 of 6 South American HCFs included in this study. The high rates of broad-spectrum antibiotic use in the HCFs may impact further emergence of antibiotic resistance. Understanding how this increase in antibiotic use compares to rates of bacterial infections during this time period is critical.

**Disclosures:**

**Jose M. Munita, MD**, BioMerieux: Grant/Research Support|MSD: Grant/Research Support|Pfizer: Grant/Research Support **Matias Salomao, MD**, Cepheid: Lecture.

**Figure 1.**
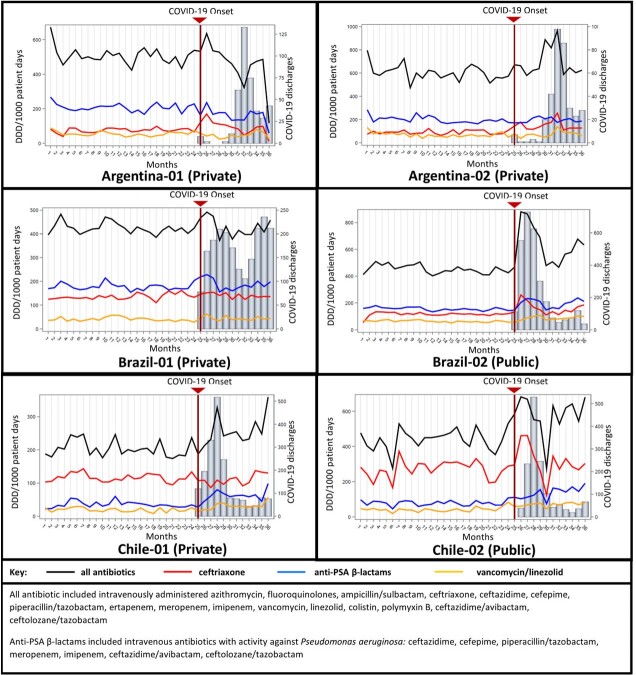
Monthly Antibiotic Use From March 2018 through February 2021 (36 months)

